# Evaluating montelukast-second-generation antihistamine combinations versus monotherapy in allergic rhinitis: A network meta-analysis

**DOI:** 10.5415/apallergy.0000000000000236

**Published:** 2026-01-13

**Authors:** Angelina Wandana, Joyceline Chika Tanely, Robby Malik Chandra Sudrajat, Jeanne Alexandra, Naja Nurilhaqqi Araz Albani, Euphemia Seto Anggraini Widyastuti, Stevent Sumantri

**Affiliations:** 1 Department of Internal Medicine, Faculty of Medicine, Universitas Pelita Harapan, Tangerang, Indonesia; 2 Department of Internal Medicine, Faculty of Medicine and Health Sciences, Universitas Katolik Indonesia Atma Jaya, Jakarta, Indonesia; 3 Department of Internal Medicine, Faculty of Medicine, Universitas Indonesia, Depok, Indonesia; 4 Department of Internal Medicine, Faculty of Medicine, Universitas Syiah Kuala, Banda Aceh, Indonesia; 5 Allergy and Clinical Immunology Division, Department of Internal Medicine, Faculty of Medicine, Universitas Pelita Harapan, Tangerang, Indonesia

**Keywords:** Allergic rhinitis, combination therapy, monotherapy, montelukast, second-generation antihistamines

## Abstract

**Background:**

Allergic rhinitis (AR) is an atopic condition affecting over 400 million people worldwide, impairing quality of life and often leading to complications such as asthma and sinusitis. Montelukast, a leukotriene receptor antagonist, is often used in combination with second-generation antihistamines (sgAHs) to enhance symptom control. However, the relative efficacy of different montelukast-sgAH combinations remains unclear.

**Objective:**

To evaluate and compare the efficacy of montelukast combined with various sgAHs versus montelukast monotherapy in patients with AR.

**Methods:**

Randomized controlled trials (RCTs) comparing montelukast-sgAH combinations to montelukast alone were identified from 5 electronic databases up to 2025. Outcomes included Total Nasal Symptom Score (TNSS; 0–12), Daytime and Nighttime Nasal Symptom Scores (DNSS, NNSS; 0–3), and Rhinoconjunctivitis Quality of Life Questionnaire (RQLQ; 0–6). Risk of bias (RoB) was assessed using Cochrane RoB 2.0. A frequentist network meta-analysis with subgroup analysis and meta-regression was performed using RStudio and Jeffreys’s Amazing Statistics Program.

**Results:**

Seventeen RCTs involving 2,655 participants were included. For TNSS improvement, montelukast + desloratadine combination is significantly better than monotherapy in adults (mean difference [MD] = −0.92 [−1.15 to −0.69]) and children (MD = −1.95 [−3.46 to −0.44]). Effects on RQLQ and DNSS were inconsistent, while NNSS improved with montelukast-levocetirizine (*P* = 0.0384). Heterogeneity was high, but most studies showed low risk of bias. No serious adverse events were reported.

**Conclusion:**

Montelukast-sgAH combinations may improve symptoms over monotherapy, especially with desloratadine in both adults and children. However, variability across outcomes and high heterogeneity warrant cautious interpretation and further research.

## 1. Introduction

Allergic rhinitis (AR) is a condition caused by atopic etiologies, with patients generally presenting with symptoms of nasal congestion, sneezing, rhinorrhea, and itching [1]. According to the Allergic Rhinitis and its Impact on Asthma (ARIA) document, the classification is based on chronicity, which could be intermittent or persistent, and severity, which is associated with the symptoms and quality of life, ranked as mild, moderate, or severe [1]. The disease is commonly associated with significant morbidity, decreased productivity, and high healthcare costs, affecting 1 in 6 individuals [1]. In accordance with a physician diagnosis, the prevalence of AR occurs approximately 15%, and is estimated to be 30% based on presenting nasal symptoms [1]. The disease has a peak incidence in the second to fourth decades of life, which continuously declines [1]. Chronic rhinitis has been reported to have a higher prevalence among adults, whereas seasonal rhinitis is more common in the children’s age group [1].

The allergic response of AR is known to have a basic mechanism of an IgE-mediated immune response [1]. The reaction is classified into early- and late-phase [1]. The early reactions involve a response against inhaled allergens, causing inflammation with its mechanisms driven by the type 2 helper (Th2) cells, in an acute onset occurring within 5 to 15 minutes of antigen exposure [1]. This causes degranulation of host mast cells, releasing histamine, a primary mediator of AR [1]. Histamine, in turn, induces the processes in which presenting symptoms of AR would normally occur [1]. Histamine stimulates the trigeminal nerve, which induces sneezing, and stimulates the mucous glands, resulting in rhinorrhea [1]. Leukotrienes and prostaglandins exert their actions on blood vessels, which causes uncomfortable nasal congestion [1]. Within the next 4 to 6 hours post the initial response, the development of the late-phase responses is initiated by cytokines [1]. These mediators facilitate infiltration of chronic immune cells, such as eosinophils, T-lymphocytes, and basophils, towards the nasal mucosa, producing nasal edema and congestion [1].

Pharmacological options include oral and intranasal H_1_ antihistamines, intranasal corticosteroids, oral and intranasal decongestants, intranasal anticholinergics, and leukotriene receptor antagonists (LTRA) [2]. Antihistamines, both first-generation and second-generation (sgAHs), are effective in the symptom control of AR [2]. Despite this, first-generation treatment options have a tendency to be more sedating due to their ability in crossing the blood-brain barrier [2]. These medications also give rise to side effects due to the stimulation of muscarinic receptors, causing urinary retention, constipation, dry mouth, and/or tachycardia [2]. The enhanced selectivity for H_1_ receptor selectivity, the longer half-lives, and most importantly, the minimum sedative effects exhibited by sgAHs render them preferable for therapeutic options [2]. Persistent Rhinitis Trial (Xyzal) was the first extensive, long-term clinical trial conducted involving 551 patients diagnosed with persistent rhinitis during the 6-month study period [3]. Levocetirizine treatment was found to enhance the quality of life, as well as to reduce the severity of symptoms experienced by individuals [3]. Following this, the XPERT (XYZAL in Persistent Rhinitis Trial) study involving 551 patients with AR exhibited an increase of patients’ quality of life, as well as relieving AR-associated symptoms [3]. Subsequently, a meta-analysis involving 18,014 participants across 48 studies showed that levocetirizine produced modest sedative effects when compared with first-generation antihistamines [3]. A meta-analysis involving 3,108 participants across 13 studies showed that desloratadine was significantly effective in reducing the severity of symptoms associated with AR [4].

Montelukast is a highly selective, orally administered cysteinyl leukotriene type-1 receptor, which specifically inhibits leukotriene D4 [4]. It is well-tolerated among both children and adult populations, demonstrating a relatively safe profile [4]. Side effects commonly reported included headache, upper respiratory infections, pharyngitis, and the incidence of otitis media, with events occurring ≥2% above placebo according to previous studies [4]. In several studies, including Meltzer et al. [5]; Nayak et al. [6]; Philip et al. [7,8]; and Van Adelsberg et al [9,10], findings show that montelukast monotherapy was significantly effective in reducing AR-associated symptom scores, which included both daytime and nighttime symptoms [4–10]. Moreover, studies involving combination therapy of montelukast with antihistamines such as loratadine or cetirizine showed increased quality of life among patients and greater efficacy towards both nighttime and daytime symptoms [4].

With the continuously evolving therapeutic options for AR, assessing the efficacy of montelukast combination therapy with sgAHs when compared with monotherapy is crucial for guiding clinical practice. Despite the multiple randomized controlled trials (RCTs) that have explored these interventions, comprehensive and direct comparisons involving all relevant therapeutic approaches remain limited. To address this gap, this study aims to perform a network meta-analysis (NMA) assessing the comparative efficacy of montelukast-sgAHs combination therapy versus monotherapy with synthesizing existing evidence in informing optimal therapeutic strategies for AR.

## 2. Materials and methods

### 2.1. Study design

This meta-analysis will be conducted following the Preferred Reporting Items for Systematic Reviews and Meta-Analyses guidelines to ensure comprehensive and standardized reporting. The study aims to quantitatively synthesize available evidence on the evaluation of the montelukast-sgAH combination and monotherapy on AR management. Peer-reviewed and nonpeer-reviewed data will be included to obtain the most evidence possible.

### 2.2. Methodology

#### 2.2.1. Network meta-analysis

To evaluate and compare the relative efficacy of multiple montelukast–antihistamine combination therapies versus montelukast monotherapy, a frequentist NMA was conducted using RStudio version 2024.12.1 + 56. NMA enables indirect comparisons between interventions that may not have been directly compared in head-to-head trials, offering a more comprehensive synthesis of the available evidence.

The NMA incorporated mean difference (MD) effect sizes with 95% confidence intervals (CIs) as summary measures, using continuous outcomes derived from standardized scoring systems such as the Total Nasal Symptom Score (TNSS; 0–12), Daytime Nasal Symptom Score (DNSS; 0–3), Nighttime Nasal Symptom Score (NNSS; 0–3), and the Rhinoconjunctivitis Quality of Life Questionnaire (RQLQ; 0–6). TNSS evaluates nasal congestion, rhinorrhea, nasal itching, and sneezing. DNSS and NNSS assess symptom burden during the day and night, respectively, with NNSS focusing on sleep disruption. RQLQ evaluates quality of life across 7 domains (activities, sleep, nasal/eye symptoms, emotional impact, etc.), with each item rated from 0 (not troubled) to 6 (extremely troubled).

To visualize and interpret the network structure and comparative efficacy, several outputs were generated. A network plot was constructed to illustrate the relationships and direct comparisons across interventions, providing a visual overview of the study connections. P-score ranking analysis, heatmap, and league tables were used to summarize all possible pairwise comparisons and rank the treatments based on their relative efficacy. Subgroup-specific forest plots were developed to depict treatment effects across different population groups and outcome measurement instruments, enabling a more nuanced understanding of intervention efficacy. Additionally, funnel plots were utilized to detect potential small-study effects or publication bias within the network.

Subgroup analyses within the NMA framework were conducted to explore variations in treatment efficacy by age (eg, children versus adults) and scoring instrument (eg, TNSS versus RQLQ). This network-level synthesis was complemented by classical meta-analysis and meta-regression techniques, providing a multidimensional and rigorous evaluation of therapeutic outcomes.

#### 2.2.2. Selection of published studies

The literature screening, data extraction, quality assessment, and statistical analyses will be conducted through a combination of manual procedures and dedicated software tools to ensure accuracy, transparency, and reproducibility throughout the research process. Zotero will be used for the blinded and independent screening of titles and abstracts, facilitating efficient inclusion and exclusion decision-making and conflict resolution between reviewers. Further table extraction was done in Microsoft Excel by 5 respective reviewers. The meta-analysis will be performed using RStudio, which allows for statistical pooling of effect sizes, evaluation of heterogeneity, and generation of forest and funnel plots. To further explore sources of heterogeneity, meta-regression and visualization through bubble plots will be conducted using Jeffreys’s Amazing Statistics Program (JASP), which offers a user-friendly interface for classical statistical analysis. This integrated approach ensures a robust methodological workflow in alignment with current best practices in evidence synthesis.

#### 2.2.3. Checking registered reviews on PROSPERO

To ensure the credibility and transparency of our study, we registered the review protocol in the International Prospective Register of Systematic Reviews (PROSPERO) (CRD420251059136). A comprehensive preliminary search was conducted using the keywords “second-generation antihistamines,” “montelukast,” “allergic rhinitis,” “combination therapy,” and “monotherapy.” A total of 18 existing systematic reviews were screened for potential overlaps in title and content. No significant similarities were identified, supporting the novelty and originality of our review.

#### 2.2.4. Inclusion and exclusion criteria

Studies were considered eligible for inclusion if they met the following criteria: (1) RCT design; (2) involved patients diagnosed with allergic-type rhinitis; (3) compared the efficacy of different montelukast-sgAH combination therapies against montelukast monotherapy; and (4) utilized standardized scoring systems such as the NNSS, DNSS, or TNSS to quantitatively assess nasal symptom improvement. Studies were excluded if they were duplicate publications, lacked retrievable full-texts, involved animal subjects, presented insufficient data, reported incomplete outcomes, or included patients diagnosed with nonallergic types of rhinitis.

#### 2.2.5. Data extraction and quality assessment

Following the inclusion criteria, 4 independent reviewers extracted relevant information from each eligible study using a standardized data extraction form. The collected data included: first author’s name and year of publication, country of origin, study design, follow-up period, participant age, type of rhinitis (eg, seasonal or perennial AR), sample size in both intervention and control groups, as well as the details of the interventions and comparators used. In addition, outcome data were extracted, including the scoring systems used to evaluate nasal symptoms, baseline and follow-up scores for both intervention and control groups, the statistical analysis method applied, and corresponding *P* values to determine efficacy. Disagreements during the extraction process were resolved through discussion or adjudication by a fifth reviewer.

To evaluate study quality, we used the Cochrane RoB (2.0) across 7 domains (random sequence generation, allocation concealment, blinding of participants and personnel, blinding of outcome assessment, incomplete outcome data, selective reporting, and other bias). Each domain was judged low, unclear, or high risk, and an overall study-level judgment was assigned by 2 independent reviewers with consensus through discussion or third-party adjudication. In addition, we assessed the certainty of evidence using the Grading of Recommendations Assessment, Development, and Evaluation (GRADE) approach for each clinically important outcome and comparison. Because the included studies were randomized trials, certainty ratings initially started at high and could be downgraded for concerns about risk of bias, inconsistency/heterogeneity, indirectness, imprecision (eg, wide CIs or not meeting optimal information size), and publication bias; for network estimates, we also considered transitivity and incoherence consistent with GRADE guidance for NMA. Final certainty ratings (high, moderate, low, or very low) and their rationales were summarized in a Summary-of-Findings table.

#### 2.2.6. Statistical analyses

The treatment effect of montelukast–antihistamine combination therapy compared with montelukast monotherapy in patients with AR was assessed using MD with 95% CIs, as the outcomes were continuous variables. When standard deviations were not directly reported, they were estimated from alternative statistics such as standard error, CI, or interquartile range following Cochrane Handbook guidance.

Heterogeneity among studies was assessed using the Cochran’s Q test and quantified using the *I*² statistic. A *P* value ≤ 0.10 or *I*² > 50% was considered indicative of substantial heterogeneity. Initially, a fixed-effects model was applied; in the presence of significant heterogeneity, a random-effects model using the DerSimonian–Laird method was employed.

Subgroup analyses were performed to explore treatment effects across different populations and outcome instruments. The subgroups analyzed included: TNSS in adults, TNSS in children, DNSS in adults, NNSS in adults, and RQLQ in adults. These subgroup comparisons were conducted using MD estimates within each subgroup to determine consistency or variation in efficacy. When appropriate, meta-regression analysis was performed using JASP version 0.19.3 to investigate whether subgroup variables moderated effect sizes, and results were visually presented using bubble plots.

## 3. Results

### 3.1. Literature search

A total of 143 records were initially identified through database searches, including PubMed (n = 10), ProQuest (n = 102), ScienceDirect (n = 25), Scopus (n = 4), and medRxiv (n = 1). These are obtained by using specific search strategy for each databases, including “(montelukast AND monotherapy AND (levocetirizine OR loratadine OR fexofenadine OR antihistamine)) AND (‘allergic rhinitis’ OR ‘persistent allergic rhinitis’) AND (‘treatment outcome’ OR ‘symptom improvement’ OR TNSS OR NNSS OR DNSS) AND (randomized controlled trial [Publication Type] OR randomized [Title/Abstract] OR RCT [Title/Abstract])” for Pubmed, “(montelukast AND monotherapy AND (levocetirizine OR loratadine OR fexofenadine OR antihistamine)) AND (‘allergic rhinitis’ OR ‘persistent allergic rhinitis’) AND (‘treatment outcome’ OR ‘symptom improvement’ OR TNSS OR NNSS OR DNSS) AND (‘randomized controlled trial’ OR RCT OR ‘clinical trial’)” for ProQuest, “montelukast AND monotherapy AND (antihistamine) AND (‘allergic rhinitis’ OR ‘persistent allergic rhinitis’) AND (TNSS OR NNSS OR DNSS OR ‘symptom score’)” for ScienceDirect and Scopus, with lastly “(Montelukast) AND (‘second generation antihistamine’) AND (‘allergic rhinitis’)” for medRxiv.

**Figure 1.**
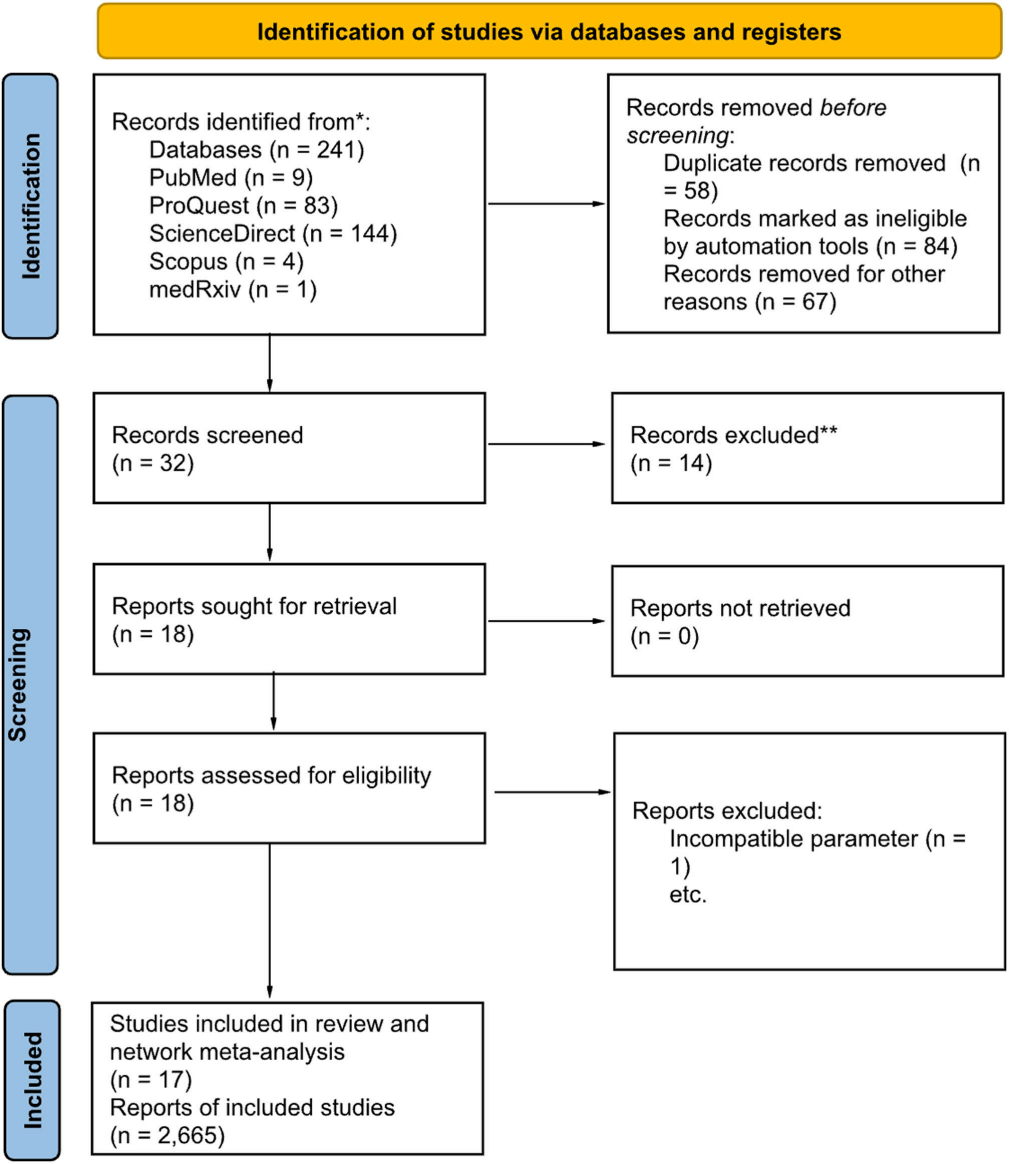
PRISMA 2020 flowchart. Preferred Reporting Items for Systematic Reviews and Meta-Analyses

After removing 111 records before screening due to duplication (n = 26), automation exclusions (n = 51), and other reasons (n = 34), 32 records remained for screening. Of these, 14 were excluded based on title and abstract review, and 18 reports were sought for retrieval, with all successfully retrieved. Upon full-text assessment of these 18 reports, 1 was excluded due to incompatible parameters. To sum up, 17 studies were included in the systematic review and NMA, representing a total of 2,665 individual reports across studies. This literature search process is summarized in Figure 1.

### 3.2. Included studies

A total of 17 RCTs involving 2,655 participants—comprising both adult and child patients with seasonal, perennial, or persistent AR—were analyzed in this study. The included studies compared the efficacy of Montelukast combined with sgAHs versus montelukast monotherapy. A summary of the key data from each included study is presented in Table 1.

**Table 1.** Key data of the included studies

Author, year	Country	Study design	Follow-up	Age (mean ± SD)	intervention	Control	Type of rhinitis	Sample size		Outcome measures	Follow-up results (mean ± SD)		Analysis method	*P*	Overall risk of bias
								I	C		I	C			
Andhale et al. (2016) [11]	India	Double-blind Randomized Controlled Trial	2 weeks	45 ± 15	Montelukast-levocetirizine fixed-dose combination	Montelukast	Allergic rhinitis	25	28	DNSS	1.5 ± 1.9	1.9 ± 2.3	*t* test	0.4916	Unclear
										NNSS	1.2 ± 2.2	1.2 ± 1.8		1	
Ciebiada et al. (2011) [12]	Poland	Double-blind Randomized Controlled Trial	42 days	28.88 ± 2.41	Montelukast-levocetirizine fixed-dose combination	Montelukast	Persistent allergic rhinitis	20	20	Congestion Score	0.6 ± 0.18	1.05 ± 0.18	*t* test	<0.001	Low
MK Kim et al. (2018) [13]	Republic of Korea	Double-blind Randomized Controlled Trial	4 weeks	43.32 ± 15.02	Montelukast-levocetirizine fixed-dose combination	Montelukast	Perennial allergic rhinitis	116	112	DNSS	−0.98 ± 0.06	−0.81 ± 0.06	*t* test	<0.001	Low
										NNSS	−0.61 ± 0.05	−0.53 ± 0.05	*t* test	<0.001	
Ciebiada et al. (2008) [14]	Poland	Double-blind Randomized Controlled Trial	6 weeks	28.88 ± 2.41	Montelukast-levocetirizine	Montelukast	Persistent allergic rhinitis	20	20	NNSS	0.54 ± 0.22	0.79 ± 0.28	*t* test	0.0033	Unclear
Panchal et al. (2020) [15]	India	Double-blind Randomized Controlled Trial	16 days	33.91 ± 11.27	Montelukast-levocetirizine	Montelukast	Seasonal allergic rhinitis	93	93	DNSS	−1.10 ± 0.056	−0.93 ± 0.053	ANOVA	<0.001	Unclear
										NNSS	−0.71 ± 0.047	−0.61 ± 0.048		<0.001	
										RQLQ	−1.34 ± 0.068	−1.17 ± 0.068		<0.001	
Ciebiada et al. (2013) [16]	Poland	Double-blind Randomized Controlled Trial	6 weeks	28.9 ± 2.7	Montelukast-levocetirizine	Montelukast	Persistent allergic rhinitis	20	20	TNSS	2.14 ± 1.74	4.99 ± 3.40	Mann Whitney’s, Welch’s *t* test	0.0024	Low
Ciebiada et al. (2013) [16]					Montelukast–desloratadine			20	20		3.04 ± 1.79	5.73 ± 2.68		0.0007	
Lu et al. (2009) [17]	United States	Double-blind Randomized Controlled Trial	2 weeks	34.0 ± 12.7	Montelukast–oratadine	Montelukast	Seasonal allergic rhinitis	174	111	TNSS	−0.43 ± 0.54	−0.31 ± 0.51	*t* test	0.0603	Low
Lu et al. (2009) [17]				32.8 ± 12.6		Montelukast		209	103		−0.38 ± 0.55	−0.34 ± 0.52		0.53	
Meltzer et al. (2000) a [5]	United States	Double-blind Randomized Controlled Trial	2 weeks	37	Montelukast–loratadine	Montelukast	Seasonal allergic rhinitis and asthma	45	95	TNSS	−0.54 ± 0.48	−0.39 ± 0.45	*t* test	0.079	Low
										RQLQ	−1.05 ± 1.23	−0.89 ± 1.07		0.454	
Meltzer et al. (2000) b [5]								45	90	TNSS	−0.54 ± 0.48	−0.31 ± 1.52		0.193	
										RQLQ	−1.05 ± 1.23	−0.68 ± 1.04		0.086	
Miao (2020) [18]	China	Double-blind Randomized Controlled Trial	2 weeks	36.49 ± 4.52	Montelukast–loratadine	Montelukast	Allergic rhinitis	40	40	TNSS	−1.78 ± 0.62	−1.33 ± 0.69	*t* test	0.003	Low
										RQLQ	−24.22 ± 10.7	−10.21 ± 13.08		0	
Nayak et al. (2002) [6]	United States	Double-blind Randomized Controlled Trial	2 weeks	36.96 ± 12.36	Montelukast–loratadine	Montelukast	Seasonal allergic rhinitis	302	155	TNSS	−0.58 ± 0.58	−0.48 ± 0.54	*t* test	0.0686	Low
										RQLQ	−1.16 ± 1.15	−1.09 ± 1.08		0.5216	
Pullerits et al. (2002) [19]	Sweden	Double-blind Randomized Controlled Trial	2 months	8.42 ± 3.18	Montelukast–loratadine	Montelukast	Seasonal allergic rhinitis	15	16	TNSS	−0.4 ± 1.53	0.3 ± 1.9	*t* test	0.2667	Low
Yildiz et al. (2019) a [20]	Turkey	Double-blind Randomized Controlled Trial	3 months	36.30 ± 7.10	Montelukast–desloratadine	Montelukast	Allergic rhinitis	40	40	TNSS	0.5 ± 0.4	1.5 ± 0.6	*t* test	<0.001	Low
Yildiz et al. (2019) b [20]				36.15 ± 5.8	Montelukast-levocetirizine dihydrochloride	Montelukast					0.5 ± 0.4	1.5 ± 0.6		<0.001	
CK Kim et al. (2024) [21]	South Korea	Double-blind Randomized Controlled Trial	4 weeks	9.73 ± 2.47	Montelukast-levocetirizine fixed-dose combination	Montelukast	Allergic rhinitis	71	76	DNSS	−1.27 ± 0.65	−1.13 ± 0.63	ANOVA	0.1874	Unclear
										NNSS	−1.1 ± 0.8	−0.87 ± 0.8		0.0837	
Ghanbari et al. (2024) [22]	Iran	Double-blind Randomized Controlled Trial	8 weeks	7.60 ± 2.87	Montelukast–desloratadine	Montelukast	Children allergic rhinitis	16	13	TNSS	4.75 ± 2.26	2.80 ± 1.90	*t* test	0.018	High
Huang et al. (2009) [23]	China	Double-blind Randomized Controlled Trial	3 weeks	9.45 ± 3.97	Montelukast–loratadine	Montelukast	Children allergic rhinitis	50	50	TNSS	−1.77 ± 0.58	−1.33 ± 0.57	*t* test	0.0002	Low
Xiao & Zhang (2008) [24]	China	Double-blind Randomized Controlled Trial	2 weeks	8.43 ± 3.18	Montelukast–loratadine	Montelukast	Children allergic rhinitis	60	60	TNSS	−1.8 ± 0.51	−1.35 ± 0.57	*t* test	1.285 × 10⁻⁵	Low
Yamamoto et al. (2012) [25]	Japan	Double-blind Randomized Controlled Trial	50 days	26.55 ± 2.30	Montelukast–loratadine	Montelukast	Seasonal allergic rhinitis	21	21	TNSS	−82.3 ± 52.11	−56.2 ± 42.4	*t* test	0.083	Low

C, control; DNSS, Daytime Nasal Symptom Scores; I. intervention; NNSS, Nighttime Nasal Symptom Scores; RQLQ, Rhinoconjunctivitis Quality of Life Questionnaire; SD, Standard Deviation; TNSS, Total Nasal Symptom Score.

Various studies included in this review have investigated the efficacy of montelukast, antihistamines (levocetirizine, desloratadine, and loratadine), and their combination in treating AR. Andhale et al. [11] reported that montelukast therapy, levocetirizine, and the combination of both have the same level of efficacy, but recommended monotherapy as it is considered safer and more effective [11]. Conversely, Ciebiada et al. [12, 14, 16] consistently found that combination therapy provided greater improvement in nasal congestion and symptom scores compared with monotherapy. Kim et al. [13] and Panchal et al. [15] also demonstrated that fixed-dose combinations (FDCs) suggest a potential for superiority in tolerability and safety compared with monotherapy. Huang et al. [23] and Yildiz et al. [20] also support the use of combination therapy, particularly in resistant or severe cases [26]. In line with this, Kim et al. [21] and Ghanbari et al. [22] legitimized that in children populations treated with montelukast–antihistamine combinations, there was better symptom control with fewer side effects [21, 22]. Although Miao [18] and Xiao and Zhang [24] prioritize findings favoring combination therapy, based on significant improvements in TNSS and RQLQ, some studies yield heterogeneous results [26]. For example, although improvements in sneezing and itching were noted in the studies by Lu et al. [17], Yamamoto et al. [25], and Nayak et al. [6], no significant advantage was found in TNSS or nasal congestion in the combination therapy group [17 5, 25]. Meltzer et al. [5] (2000a, 2000b) and Pullerits et al. [19] also noted that the significance of differences in TNSS or RQLQ overall was inconsistent, although combination therapy still improved individual symptoms such as sneezing and itching [5, 19]. Thus, combination therapy has better efficacy, particularly in nasal congestion and sneezing. However, the added value of combination therapy compared with monotherapy is conditional on the population, symptom profile, and study design.

Risk of bias. Using the RoB 2.0, most trials were judged at low risk across the 7 domains. Several studies—such as those by Ciebiada et al. [12, 14, 16], Meltzer et al. [5], Pullerits et al. [19], and Yamamoto et al [25]—were consistently reported low risk. Allocation concealment was the domain most frequently rated unclear owing to insufficient reporting in a few trials (eg, Andhale et al [11] 2016; Kim et al. [21]; Panchal et al. [15]). One study (Ghanbari et al. [22]) was judged high risk for performance bias due to the lack of blinding of participants and personnel. No systematic high-risk concerns were identified for detection, attrition, reporting, or other bias domains. The study-level summary and the domain-level distribution are shown in Supplementary Figure X1-A https://links.lww.com/PA9/A78 and Supplementary Figure X1-B https://links.lww.com/PA9/A79, respectively.

### 3.3. Subgroup analyses, sensitivity analyses, league table

Subgroup analyses, sensitivity analyses, and league tables were conducted for each outcome measure in this study.

#### 3.3.1. Total nasal symptom score (TNSS; 0–12)

For TNSS, subgroup analyses for the adult and children population were done to evaluate potential differences in treatment efficacy based on age group.

##### 3.3.1.1. Adult population

**Figure 2.**
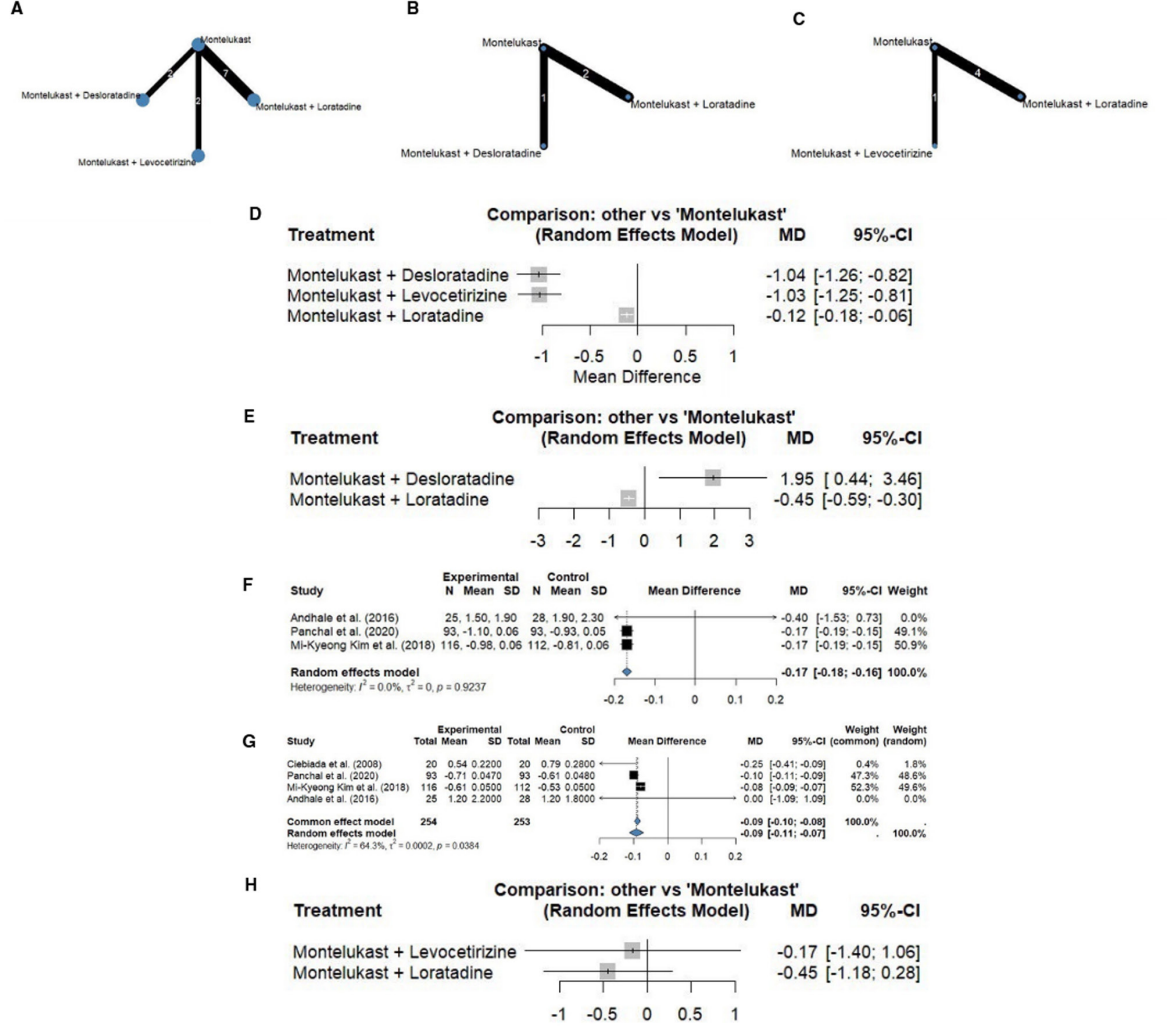
(A–C) Network Plots: TNSS in adults, TNSS in children, RQLQ in adults. (D–H) Forest plots: TNSS in adults, TNSS in children, DNSS in adults, NNSS in adults, RQLQ in adults. DNSS, Daytime Nasal Symptom Scores; NNSS, Nighttime Nasal Symptom Scores; RQLQ, Rhinoconjunctivitis Quality of Life Questionnaire; TNSS, Total Nasal Symptom Score.

**Figure 3.**
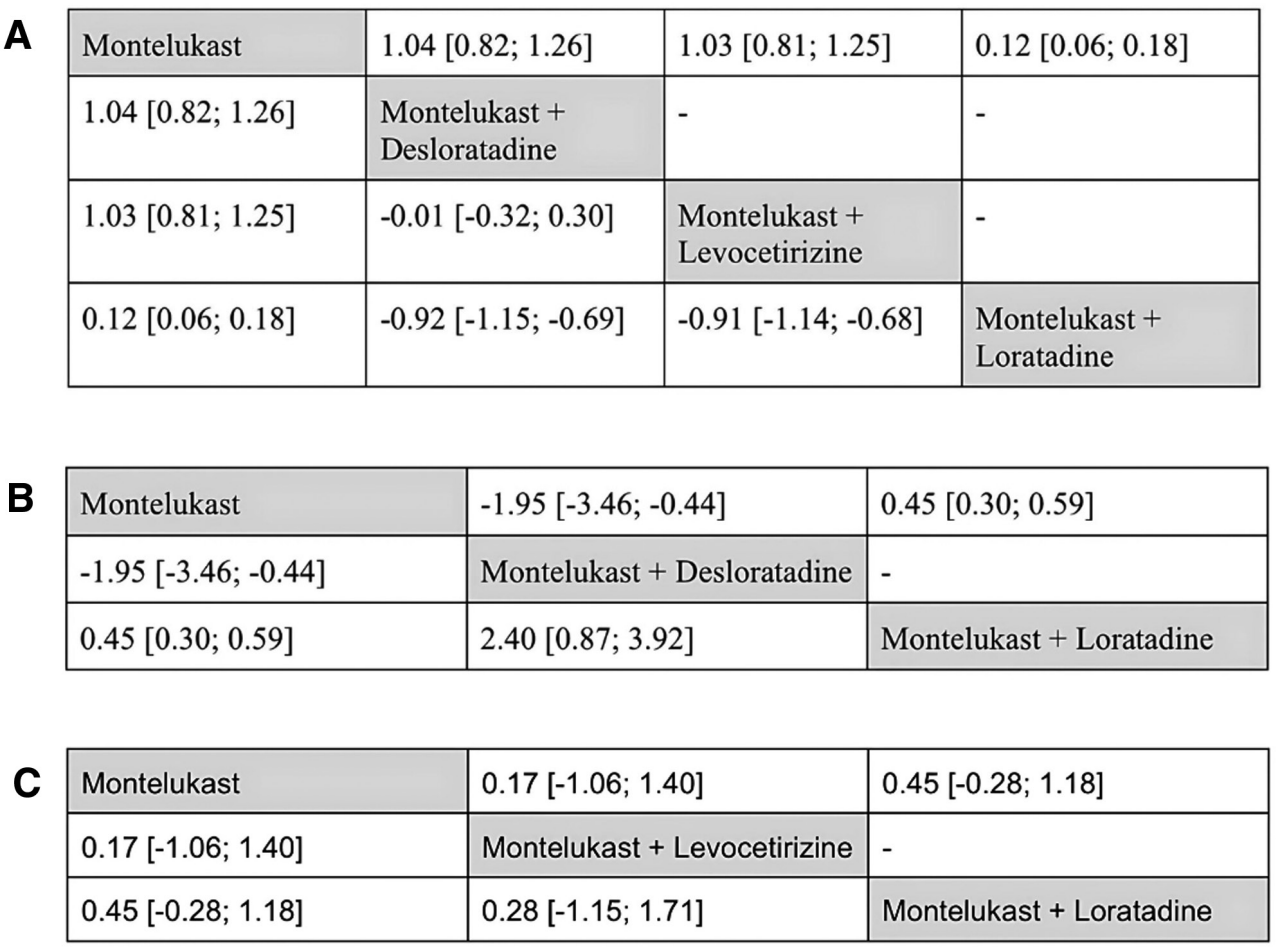
League tables comparing treatments across outcomes: (A) TNSS in adults, (B) TNSS in children, (C) RQLQ in adults. RQLQ, Rhinoconjunctivitis Quality of Life Questionnaire; TNSS, Total Nasal Symptom Score.

The NMA suggested that the combination of montelukast with sgAHs, such as desloratadine, levocetirizine, or loratadine, was associated with statistically significant improvements in clinical outcomes among adult patients compared with montelukast monotherapy. Symptom severity was measured using the TNSS, a scale ranging from 0 to 12, where higher scores reflect greater nasal symptom burden. All 3 combination therapies produced statistically significant improvements in TNSS scores compared with montelukast alone, indicating clinically relevant treatment effects. The network plot is depicted in Figure 2A, while the forest plot is provided in Figure 2D. The league table ranking in Figure 3A shows that montelukast combined with desloratadine and montelukast combined with levocetirizine provided the greatest improvements in TNSS compared with montelukast monotherapy, with statistically significant MDs of −0.92 (95% CI, −1.15 to −0.69; *P* < 0.001) and −0.91 (95% CI, −1.14 to −0.68; *P* < 0.001), respectively. Montelukast combined with loratadine also demonstrated a significant benefit over montelukast alone, with an MD of 0.12 (95% CI, 0.06–0.18; *P* < 0.001). These findings indicate that combination therapy consistently outperformed montelukast monotherapy in adults.

While the analysis indicated potential benefits of certain interventions, it also revealed substantial heterogeneity. The between-study variance was considerable (τ² = 0.1196; τ = 0.3459), and the inconsistency index (*I*² = 92.0%) suggests that most of the variation in treatment effects arose from differences across studies rather than random error. This high heterogeneity (*I*² > 75%) may be influenced by factors such as study design, patient characteristics, intervention protocols, or regional differences, including studies conducted in Asia, America, and Europe. These findings highlight the need for cautious interpretation.

##### 3.3.1.2. Children population

In the children population, an NMA evaluating the efficacy of different treatment combinations on TNSS revealed that the combination of montelukast and desloratadine provided significantly greater symptom relief compared with both montelukast alone and montelukast combined with loratadine. Notably, montelukast alone was also more effective than the montelukast–loratadine combination. The network plot is presented in Figure 2B, while the forest plot is presented in Figure 2E.

Results from the league table ranking, as seen in Figure 3B, provide further insight into comparative efficacy. The combination of montelukast plus desloratadine versus montelukast alone showed an MD of –1.95 with a 95% CI of –3.46 to –0.44 and a *P* value of 0.0114, indicating a statistically significant and clinically meaningful reduction in TNSS. A negative MD suggests better symptom control with the combination therapy. Conversely, the comparison of montelukast plus loratadine versus montelukast alone yielded a positive MD of 0.45 (95% CI, 0.30–0.59; *P* < 0.001), indicating a significantly worse TNSS outcome when loratadine was added to montelukast.

The statistical heterogeneity in the analysis was substantial, with τ² = 0.0841 and τ = 0.2900, indicating notable variability in treatment effects across studies. The *I*² value was 79.0% (95% CI, 22.6%–99.5%), suggesting that approximately 79% of the variability in effect sizes was due to true differences between studies rather than random chance.

#### 3.3.2. Daytime nasal symptom score (0–3)

A meta-analysis evaluating the DNSS (scale 0–3) showed that treatment with montelukast combined with levocetirizine did not provide a significant advantage over montelukast alone in adults with AR. The forest plot is illustrated in Figure 2F. The MD was 0.17, with a 95% CI of –1.06 to 1.40 and a *P* value of 0.9237, indicating a statistically nonsignificant result. The wide CI crossing zero and the high *P* value suggest that the 2 treatments were essentially equivalent in terms of daytime nasal symptom control. Notably, the analysis demonstrated zero heterogeneity (*I*² = 0%), indicating that the results across the included studies were highly consistent.

#### 3.3.3. Nighttime nasal symptom score (0–3)

Montelukast with levocetirizine was associated with a modest reduction on the NNSS (scale 0–3) compared with montelukast monotherapy. The MD was –0.09, with a 95% CI ranging from –0.11 to –0.07 (*P* = 0.0384), indicating a statistically significant but small improvement in nighttime nasal symptom severity. The forest plot is depicted in Figure 2G. This modest but significant improvement was observed with moderate heterogeneity across studies (*I*² = 64.3%), indicating some variability in treatment responses and suggesting that findings should be interpreted with caution.

#### 3.3.4. Rhinoconjunctivitis quality of life questionnaire (0–6)

Based on the results of the NMA, the combination of montelukast with sgAHs showed a possible trend toward improved symptom control compared to montelukast monotherapy in adult patients with AR. Symptom severity was assessed using the RQLQ, which ranges from 0 to 6, with lower scores indicating milder symptoms and better quality of life. Differences between montelukast–levocetirizine and montelukast alone, as well as montelukast–loratadine and montelukast alone, were not statistically significant in adults (τ² = 0.3906; τ = 0.6250; *I*² = 89.5% [75.9%–95.4%]), and the high heterogeneity substantially limits the confidence in these comparisons. The network plot is shown in Figure 2C, while the forest plot is illustrated in Figure 2H. According to the league table ranking as seen in Figure 3C, montelukast–levocetirizine had the highest rank with an MD of 0.17 points (95% CI, –1.06 to 1.40; *P* = 0.7856), followed by montelukast–loratadine with an MD of 0.45 (95% CI, –0.28 to 1.18; *P* = 0.2260). These rankings were not statistically significant and should be interpreted cautiously, given the broad CI and lack of consistent effects.

### 3.4. P-score ranking analysis

In the *P* score ranking analysis, montelukast–desloratadine showed the highest possibility of being the most effective intervention (*P* score = 0.735), followed by montelukast–loratadine (*P* score = 0.584). Montelukast monotherapy ranked lower (*P* score = 0.432), while Montelukast–Levocetirizine exhibited the least favorable performance (*P* score = 0.248). Visual inspection of the heatmap, as seen in Supplementary Figure X2 https://links.lww.com/PA9/A79, illustrates a gradient from green to orange, supporting the relative superiority of montelukast–desloratadine combinations compared with other treatment strategies.

### 3.5. Meta-regression, sensitivity analysis, funnel plot

The meta-regression results explored how publication year, follow-up period, and intervention sample size moderated treatment effects across different outcome measures and age groups. For TNSS in adults, the follow-up period was significantly associated with treatment effect (Estimate = −0.012, *P* < 0.001), indicating that longer follow-up was linked to smaller effect sizes. However, publication year (*P* = 0.109) and intervention sample size (*P* = 0.106) were not significant moderators. In children, none of the moderators significantly influenced TNSS, though trends were observed. For DNSS in adults, no moderator showed a significant effect, with all *P* values > 0.95. Similarly, for NNSS in adults, results were nonsignificant, although the intervention sample size approached significance (*P* = 0.066). Lastly, in the case of the RQLQ in adults, none of the moderators demonstrated statistically significant effects, with wide CIs reflecting high uncertainty. Overall, the follow-up period appeared to be the only consistent and significant moderator, particularly for adult TNSS outcomes. These results can be shown in Table 2.

**Table 2.** Meta-regression summary for each outcome measures

Parameter	Moderator	Estimate	Standard error	*t*	df	*P*	95% CI	
							Lower	Upper
TNSS adult	Publication year	−0.039	0.022	−1.778	9	0.109	−0.088	0.011
	Follow-up period (days)	−0.012	0.002	−6.27	9	<0.001	−0.016	−0.008
	Intervention sample size	0.003	0.002	1.794	9	0.106	−9.018 × 10^−4^	0.008
TNSS children	Publication year	0.14	0.043	3.277	1	0.189	−0.402	0.682
	Follow-up period (days)	0.054	0.02	2.672	1	0.228	−0.204	0.312
	Intervention sample size	−0.052	0.021	−2.451	1	0.247	−0.324	0.219
DNSS adult	Publication year	6.571 × 10^−5^	0.002	0.029	1	0.981	−0.028	0.029
	Follow-up period (days)	4.904 × 10^−6^	3.746 × 10^−4^	0.013	1	0.992	−0.005	0.005
	Intervention sample size	1.316 × 10^−5^	1.946 × 10^−4^	0.068	1	0.957	−0.002	0.002
NNSS adult	Publication year	0.012	0.006	2.128	2	0.167	−0.012	0.036
	Follow-up period (days)	−0.005	0.003	−1.432	2	0.288	−0.019	0.009
	Intervention sample size	0.001	2.799 × 10^−4^	3.687	2	0.066	−1.724 × 10^−4^	0.002
RQLQ adult	Publication year	−0.31	0.259	−1.199	3	0.317	−1.135	0.514
	Follow-up period (days)	1.531	3.631	0.422	3	0.702	−10.025	13.087
	Intervention sample size	0.016	0.028	0.577	3	0.605	−0.074	0.106

CI, confidence interval; df, degrees of freedom; DNSS, Daytime Nasal Symptom Scores; I. intervention; NNSS, Nighttime Nasal Symptom Scores; RQLQ, Rhinoconjunctivitis Quality of Life Questionnaire; SE: standard error; t: t-statistic; TNSS, Total Nasal Symptom Score.

**Figure 4.**
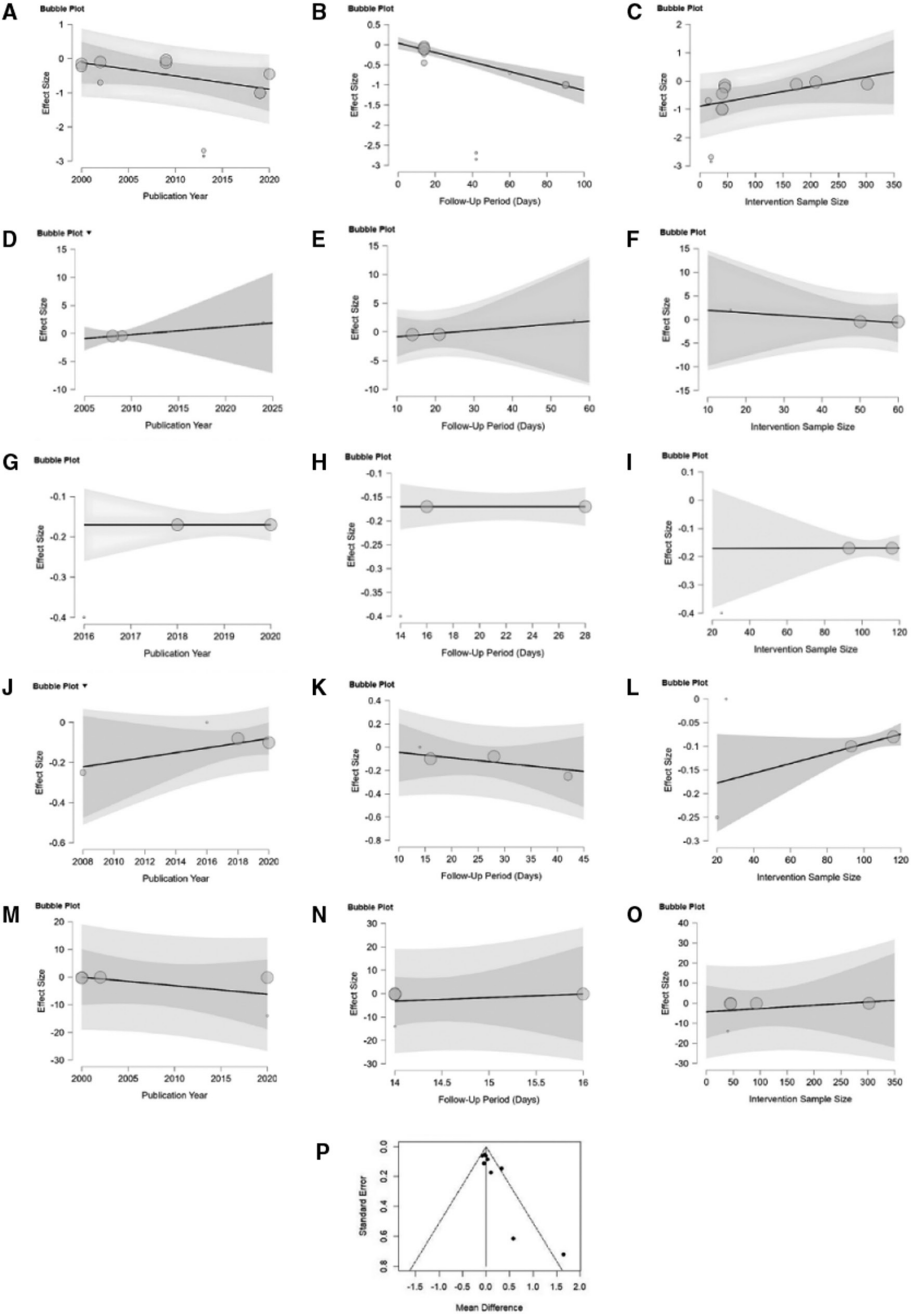
(A–C) Bubble plots for TNSS (adults) by publication year, follow-up period, and intervention sample size, (D–F) Bubble plots for TNSS (children) by the same variables; (G–I) Bubble plots for DNSS (adults); (J–L) for NNSS (adults); (M–O) for RQLQ (adults); (P) Funnel plots for TNSS (adults). DNSS, Daytime Nasal Symptom Scores; NNSS, Nighttime Nasal Symptom Scores; RQLQ, Rhinoconjunctivitis Quality of Life Questionnaire; TNSS, Total Nasal Symptom Score.

Bubble plot of publication year, follow-up period (days), and intervention sample size meta-regression are presented in Figure 4A–O. In the adult TNSS analysis, Ciebiada et al. [16] were identified as an outlier. Visual inspection of Figure 4A–C supports the significant negative association with follow-up period and the nonsignificant trends with publication year and intervention sample size. In the children’s TNSS analysis, Ghanbari et al. [22] emerged as a consistent outlier across all moderator models. Although no moderators reached significance, visual trends in Figure 4D–F suggest possible positive associations with publication year and follow-up, and a negative trend with sample size. For adult DNSS, moderator effects were negligible with no notable outliers, as shown in Figure 4G–I. In adult NNSS, Ciebiada et al. [14] appeared as an influential outlier. Though all associations were nonsignificant, the intervention sample size approached significance (*P* = 0.066), as depicted in Figure 4J–L. Lastly, for RQLQ in adults, none of the moderators were significantly associated with the effect size. Miao [18] consistently deviated from regression patterns but did not substantially influence overall results, as shown in Figure 4M–O.

Egger’s test in the TNSS adult analysis indicated a statistically significant intercept estimate of –1.8970 (SE = 0.3970, *P* = 0.0010). Visual inspection of the funnel plot also showed asymmetry, as seen in Figure 4P, suggesting the possibility of publication bias.

## 4. Discussion

### 4.1. Mechanism of the interventional drugs

The concurrent use of montelukast and sgAHs provides superior symptom control in AR by targeting distinct yet complementary inflammatory pathways. Montelukast acts as a selective antagonist of cysteinyl leukotriene type-1 (CysLT_1_) receptors, thereby inhibiting leukotriene-mediated responses such as mucosal inflammation, nasal congestion, mucus hypersecretion, and recruitment of eosinophils during the allergic cascade [10, 27]. This mechanism contributes to a reduction in airway edema and mitigates late-phase allergic inflammation.

In contrast, sgAHs exert their effect by selectively blocking histamine H_1_ receptors, which helps alleviate early-phase symptoms like pruritus, sneezing, and rhinorrhea [28].

By addressing both histamine- and leukotriene-mediated pathways, this pharmacological combination provides comprehensive relief across both early and late phases of the allergic response. Evidence from clinical trials demonstrates that such a combination significantly improves nasal symptoms, decreases biomarkers of eosinophilic inflammation, and leads to measurable improvements in patient-reported outcomes, including sleep quality and overall quality of life [10, 28–30].

Additionally, this dual therapy may offer efficacy comparable to intranasal corticosteroids in certain patient populations, representing a valuable oral treatment alternative. It has been shown to have a favorable safety profile, with adverse event rates similar to those observed with monotherapy or placebo [29]. Importantly, the combined approach is particularly advantageous for patients who suffer from both asthma and AR, given montelukast’s established efficacy in managing both conditions [10].

### 4.2. Comparison of first- and second-generation antihistamines

Antihistamines are commonly used agents in the management of allergic diseases and are broadly categorized into first-generation and second-generation based on their pharmacological properties. Both generations act by blocking H_1_ histamine receptors to prevent histamine-induced effects such as increased vascular permeability, bronchoconstriction, and smooth muscle contraction [32, 33]. However, they differ substantially in their pharmacokinetics, side effect profiles, and clinical utility [33, 32].

First-generation antihistamines, such as diphenhydramine, chlorphenamine, clemastine, promethazine, and hydroxyzine, are characterized by their ability to readily cross the blood-brain barrier. This results in significant central nervous system (CNS) effects, most notably sedation and drowsiness, which can limit their use in certain populations such as children, the elderly, and individuals requiring high levels of alertness [32, 33]. Their anticholinergic side effects—including dry mouth, blurred vision, urinary retention, and constipation—further restrict their long-term applicability [32, 33]. While effective for acute allergic reactions, motion sickness, and insomnia, they generally require multiple daily dosing due to their shorter half-lives (approximately 4–6 hours) [32]. Consequently, they are less favored for chronic allergy management and are mainly reserved for short-term symptom control or nighttime use when sedation is not a drawback [33, 34].

In contrast, sgAHs such as cetirizine, loratadine, desloratadine, fexofenadine, levocetirizine, bilastine, and rupatadine demonstrate high selectivity for peripheral H_1_ receptors and minimal CNS penetration, resulting in low sedation potential [34, 35]. These agents offer a more favorable safety profile and are thus recommended as first-line therapy for AR, chronic urticaria, and other dermatologic allergic conditions [33, 35]. Their longer duration of action supports once-daily dosing, improving adherence and convenience for patients. Although cetirizine and levocetirizine may occasionally cause mild drowsiness, they are generally well-tolerated, with the most common side effects being headache, fatigue, dry mouth, and gastrointestinal discomfort [33, 31, 36].

Evidence from NMA supports the superior performance of certain sgAHs. In AR, rupatadine (especially at a 20 mg dose) appears to be among the most effective, while levocetirizine and cetirizine also demonstrate robust efficacy. For chronic urticaria, cetirizine and levocetirizine are widely used, and dose escalation (up to 4 times the standard dose) has shown benefit in refractory cases without a significant increase in adverse effects. Unlike first-generation agents, sgAHs do not produce meaningful anticholinergic effects and are considered safer for long-term management, particularly in vulnerable populations such as children and the elderly [13, 31, 37].

The clinical decision between first-generation antihistamines and sgAHs should consider both efficacy and safety profiles. While first-generation antihistamines may offer rapid relief in acute scenarios, their CNS and anticholinergic adverse effects make them unsuitable for sustained use. On the other hand, sgAHs are strongly favored for chronic and long-term treatment of allergic diseases due to their minimal sedation, once-daily dosing, and excellent tolerability. Their use is especially appropriate in patients requiring optimal daily functioning, such as students, drivers, and those operating machinery.

### 4.3. Clinical applicability

FDCs of montelukast and sgAHs or sgAHs (levocetirizine, desloratadine, and loratadine) have been clinically available and marketed after undergoing phase III trials in several countries, such as India, Korea, Mexico, and European countries. The FDC has been reported to have better efficacy and tolerability compared with monotherapy [13, 29, 38, 39]. For example, a phase III multicentre study in Korea reported that the combination of montelukast (10 mg) and levocetirizine (5 mg) significantly reduced nasal symptom scores compared with monotherapy (*P* = 0.045) [36]. Similar findings were reported in Mexico, where the combination of montelukast (10 mg) and desloratadine (5 mg) was beneficial in improving persistent rhinitis [29]. These findings align with the 2019 ARIA Care Pathways Guidelines, which recommend combination therapy with LTRA (such as montelukast) and antihistamines for patients unresponsive to monotherapy [38, 40].

Pharmacokinetic and compliance considerations indicate bioequivalence between FDC formulations and separate administration of components, thereby facilitating once-daily treatment [41]. Tolerability analysis also showed similar safety profiles between FDC and monotherapy with levocetirizine or montelukast alone [41]. The availability of combination therapies such as montelukast–levocetirizine in various countries, supported by international guidelines, makes them a potential option for implementation in various healthcare systems, particularly in countries with limited access, where improving compliance and reducing the economic burden on patients are important considerations [42].

Beyond pharmacokinetics and compliance, safety considerations are equally important. Adverse events (AEs) associated with montelukast–antihistamine combination therapy varied across the included RCTs. Andhale et al. [11] reported that the montelukast–levocetirizine combination was associated with headache, dizziness, and dry mouth, side effects that were attributed mainly to levocetirizine. In contrast, Ciebiada et al. [12, 14, 16], Ghanbari et al. [22], Huang et al. [23], Lu et al. [17], Pullerits et al. [19], Xiao and Zhang [24], Yamamoto et al. [25], and Yildiz et al. (2019a, 2019b) did not provide specific reports of AE. Kim et al. [13] found that treatment-emergent AE (TEAEs) occurred in 16.67% of subjects in the combination group and 16.36% in the montelukast group, with upper respiratory tract infection, nasopharyngitis, and tonsillitis being the most common in the combination group, while upper respiratory tract infection and pruritus were noted in the monotherapy group. Two serious AE (SAEs), gastric cancer in the combination group and cholelithiasis in the montelukast group, were reported but were not attributed to the study drugs. Similarly, Panchal et al. [15] observed that the fixed-dose montelukast–levocetirizine combination was generally well-tolerated, with AEs reported in 17.2% of the combination group, 9.7% of the montelukast group, and 14.0% of the levocetirizine group; most AEs were mild, with hypersomnia and sedation-related symptoms attributed to levocetirizine. No deaths, SAEs, or discontinuations were reported in this study. Kim et al. [21] reported a lower incidence of AEs in the combination group (5.6%) compared with montelukast monotherapy (15.8%), with most events being mild; however, two SAEs (headache and aspiration pneumonia) occurred in the montelukast group, with no significant differences in laboratory or vital signs between groups. In earlier studies, Meltzer et al. [5] and Nayak et al. [6] noted commonly reported AEs, including headache, upper respiratory tract infections, dry mouth, asthenia, fatigue, tachycardia, and pruritus, though these were not considered severe. Miao [18] and several other studies did not report safety data. Overall, the combination therapy appeared safe and generally well-tolerated, with most AEs being mild to moderate and rarely leading to treatment discontinuation.

While safety profiles are reassuring, efficacy outcomes highlight the need for a tailored approach. Although international guidelines such as ARIA and actual global data favor combination therapy, especially for patients who do not respond to monotherapy, the results of this study indicate that the efficacy of the combination of montelukast with sgAHs is highly dependent on the type of antihistamine used and the characteristics of the population. The montelukast–desloratadine combination yields the best outcomes in adult patients as well as in children, though with high heterogeneity across studies. The findings underscore the importance of implementing the concept of personalized medicine in clinical practice, where individualized approaches must be considered. Determining the preferred drug combination should be based on an analysis of age, initial response to therapy, patient symptom profile, and other unique and personalized conditions. In this context, a measured trial-and-error strategy, while still adhering to clinical guidelines, is important to achieve optimal symptom control without burdening patients pharmacological

### 4.4. Strengths and limitations of the study

This study represents the first systematic review and NMA comparing montelukast-sgAH combinations with monotherapy in the treatment of AR, thereby addressing a critical gap in the current literature. The inclusion of multinational studies conducted across diverse populations enabled the comparative ranking of various montelukast-sgAH combinations, enhancing the generalizability of the findings. The exclusion of participants with severe respiratory comorbidities contributed to a more homogeneous study population, thereby increasing internal validity. Most of the included RCTs enrolled participants with moderate to severe AR (nasal congestion score ≥2), enabling a focused assessment of clinically relevant cases. Subgroup analyses stratified by age (children versus adults) further strengthened the findings by identifying potential differences in treatment response across demographic groups. Additionally, the consistent use of outcome measures across studies supported the robustness and comparability of the data synthesis.

At the same time, several methodological considerations are important for contextualizing these results. For DNSS results, the low heterogeneity of 0% means that any variation in the outcomes was likely due to random chance rather than differences in study design, populations, or interventions. Although this strengthens the confidence in the conclusion, it also reinforces that combining levocetirizine with montelukast does not appear to enhance daytime symptom relief in the adult population. However, a high degree of heterogeneity was observed across the other outcomes (*I*² = 92% for adult TNSS, 79% for children TNSS, 64.3% for NNSS, and 89.5% for RQLQ). While this variability was statistically accommodated using a random-effects model, it may reflect meaningful differences in study design, patient populations, intervention protocols, and geographical settings, including studies conducted in Asia, America, and Europe. This highlights the complexity of synthesizing evidence across diverse contexts and emphasizes the importance of interpreting treatment effects within their clinical and methodological frameworks.

Sensitivity analysis was done to explore the source of heterogeneities across the parameters and to assess the reliability of the findings. For TNSS in the adult population, excluding Ciebiada et al. [16] reduced heterogeneity to 26.5%. This may be explained by the fact that both studies included 100% Caucasian samples, which could have contributed to their outlier status. Differences in AR prevalence across racial groups, influenced by environmental and lifestyle factors, may underlie the increased heterogeneity. Taken together, although montelukast combined with sgAHs showed improved symptom control compared with monotherapy, particularly with desloratadine or levocetirizine, the high degree of heterogeneity warrants cautious interpretation of the pooled effect estimates. For TNSS in the children population, when Ghanbari et al. [22] were excluded, the network became disconnected, reducing heterogeneity to 0%. However, this exclusion limited comparisons to only montelukast–loratadine versus montelukast, as Ghanbari et al. [22] was the sole study comparing montelukast–desloratadine versus montelukast within the three-study NMA for TNSS in children. Therefore, the initially observed high heterogeneity is likely attributable to the small sample sizes in the included children trials, which can introduce greater variability in results. Next, for NNSS, sensitivity analysis revealed that excluding the studies by Kim et al. [13] and Ciebiada et al. [14] reduced heterogeneity to 54.1% and 53.7%, respectively. This reduction suggests that these 2 studies contributed substantially to the observed variability, possibly due to differences in study design or the type of rhinitis examined (eg, seasonal versus persistent). Although the observed effect was modest, the general direction of benefit suggests that the montelukast–levocetirizine combination may offer potential advantages in reducing nighttime nasal symptoms in the adult population with AR. However, this interpretation should remain cautious due to the variability across studies and relatively small effect sizes. Lastly, for the RQLQ results, excluding Miao [18]—the only study that investigated general AR—led to a substantial reduction in heterogeneity from 89.5% to 0%. In contrast, the other 4 studies in the analysis specifically investigated seasonal AR, and excluding any of them individually did not significantly reduce heterogeneity. This suggests that the initially high heterogeneity was largely driven by the inclusion of Miao [18], likely due to differences in study populations. Additionally, small sample sizes in some trials may have influenced result stability and contributed to wider CIs.

Pooled analyses suggest that the combination of montelukast and desloratadine may improve AR symptoms compared with monotherapy, particularly in adults, as reflected in TNSS outcomes. However, these findings should be interpreted with caution due to substantial heterogeneity across studies. Within the children’s TNSS analysis, the combination of montelukast and desloratadine consistently provided greater symptom relief than both monotherapy and the montelukast–loratadine regimen. Notably, the latter not only failed to confer additional benefit in the children population but was associated with poorer nasal symptom outcomes than montelukast alone, which is likely attributable to small sample sizes or differences in study design rather than a true inverse clinical effect. This result may be partially explained by loratadine’s relatively weaker and shorter-acting H_1_-antagonist effect compared to other sgAHs, potentially contributing to a suboptimal response in certain individuals [26]. The inclusion of the study by Ghanbari et al. [22], which used a montelukast–desloratadine combination, was necessary to preserve network connectivity in the children TNSS analysis, as the other two studies used montelukast–loratadine. Although the study added methodological complexity that likely contributed to the high heterogeneity observed in sensitivity analyses, it also improved the scope of the NMA by capturing a wider range of therapeutic approaches, even though this made the overall findings harder to interpret due to greater differences in study design and treatment protocols.

Additionally, publication bias identified for adult TNSS (Egger’s test, *P* = 0.0010) suggests a potential underrepresentation of smaller studies with null or negative findings. This may lead to an overestimation of treatment effects in the pooled analysis, as studies showing no benefit are less likely to be published and included. Consequently, the overall effect estimates should be interpreted with caution, considering the possibility of selective reporting.

Moreover, while improvements in TNSS consistently favor combination therapy, the effects on other clinically meaningful outcomes remain inconclusive due to high heterogeneity, as well as variability in statistical significance across different parameters. The variability in findings across TNSS, NNSS, DNSS, and RQLQ, where TNSS (in both adult and children populations) and NNSS showed significant results while DNSS and RQLQ did not, may reflect differences in measurement timing, instrument responsiveness, and clinical sensitivity. Daytime measures like DNSS may be more variable or less reliably reported, while RQLQ captures broader quality-of-life impacts that often require longer treatment or greater clinical improvement to demonstrate meaningful change. Variability across these outcomes may also stem from differences in baseline severity, follow-up duration, and sample size.

Finally, the limited number of antihistamines studied in combination with montelukast reflects the early phase of research in this area. Likewise, several studies employed treatment durations of 2 to 7 weeks, which may be shorter than guideline-recommended evaluation periods. Together, these observations underscore the opportunity for future high-quality RCTs to explore longer-term outcomes, incorporate a broader range of antihistamines, and evaluate the broader impact of combination therapy across diverse populations. Such studies are essential to strengthen and extend the current evidence base, identify the most effective therapeutic regimens, and support a personalized, symptom-targeted approach in both research and clinical decision-making.

## 5. Conclusion and recommendations

The current evidence tentatively suggests that the combination of Montelukast with sgAHs may be associated with improved outcomes compared with monotherapy in the management of AR. This potential benefit is supported by observed improvements in the TNSS, which encompasses key nasal symptoms such as congestion, sneezing, nasal itching, and rhinorrhea. However, these findings should be interpreted with caution, given the substantial heterogeneity across studies. Available data also indicate a generally favorable safety profile for combination therapy across both children and adult populations. Among the regimens evaluated, montelukast combined with desloratadine appears to be associated with the most improved outcomes in adults and children. However, this children’s finding is based on a single study, which limits the reliability of this conclusion. These patterns suggest a potential age-related variation in treatment response, though further evidence is needed to substantiate this observation.

Despite these encouraging trends, the existing literature lacks diversity in study populations. Future research should aim to include underrepresented groups such as pregnant individuals, patients with comorbid asthma, current smokers, and those sensitized to seasonal aeroallergens, including grass, tree, and weed pollen. Inclusion of these populations would enhance the external validity and generalizability of treatment recommendations.

Moreover, while improvements in TNSS consistently favor combination therapy, the effects on other clinically meaningful outcomes remain inconclusive due to high heterogeneity, as well as variability in statistical significance across different parameters. The variability in findings across TNSS, NNSS, DNSS, and RQLQ, where TNSS (in both adult and children populations) and NNSS showed significant results while DNSS and RQLQ did not, may reflect differences in measurement timing, instrument responsiveness, and clinical sensitivity. Daytime measures like DNSS may be more variable or less reliably reported, while RQLQ captures broader quality-of-life impacts that often require longer treatment or greater clinical improvement to demonstrate meaningful change. Variability across these outcomes may also stem from differences in baseline severity, follow-up duration, and sample size. Additional high-quality RCTs are needed to evaluate the broader impact of combination therapy and to identify the most effective regimens across diverse demographic and clinical subgroups. A personalized, symptom-targeted approach should be prioritized in future research and clinical decision-making.

## Acknowledgements

This research would not have been possible without the invaluable guidance and supervision provided by the Allergy and Clinical Immunology Division, Department of Internal Medicine, Faculty of Medicine, Universitas Pelita Harapan, Tangerang, Indonesia. We are also profoundly thankful for the constant encouragement and support from our families and friends, whose presence sustained us throughout this journey. Above all, we offer our deepest gratitude for the unending grace and blessings of Almighty God, which made this work possible.

## Conflicts of interest

The authors have no financial conflicts of interest.

## Author contributions

Conceptualization and design: Angelina Wandana. Preliminary work and preparation: Angelina Wandana, Joyceline Chika Tanely, Robby Malik Chandra Sudrajat, Jeanne Alexandra, Naja Nurilhaqqi Araz Albani. Writing and methodological development: Jeanne Alexandra, Robby Malik Chandra Sudrajat, Joyceline Chika Tanely, Angelina Wandana, Naja Nurilhaqqi Araz Albani. Analysis and interpretation: Angelina Wandana, Naja Nurilhaqqi Araz Albani. Manuscript drafting and review: Angelina Wandana, Joyceline Chika Tanely, Robby Malik Chandra Sudrajat, Jeanne Alexandra. Finalization and editing: Angelina Wandana, Joyceline Chika Tanely, Robby Malik Chandra Sudrajat, Jeanne Alexandra, Naja Nurilhaqqi Araz Albani. Supervision and guidance: Dr. Stevent Sumantri, SpPD, K-AI, DAA, FINASIM; Dr. Euphemia Seto Anggraini Widiastuti, SpPD, FINASIM.
